# Investigating the role of inorganic phosphate in tumor metabolism and metastasis

**DOI:** 10.1186/2049-3002-2-S1-P55

**Published:** 2014-05-28

**Authors:** Cesar Perez Ramirez, Dorothea Fiedler

**Affiliations:** 1 Department of Molecular Biology, Princeton University, Princeton, New Jersey, USA; 2 Department of Chemistry, Princeton University, Princeton, New Jersey, USA

## Background

Altered cell metabolism is regarded as a hallmark of cancer with numerous studies highlighting glucose and glutamine as critical nutrients for tumor proliferation. Rapidly dividing cells should also require a continuous supply of phosphate, an essential nutrient for the synthesis of nucleic acids, phospholipids and high energy metabolites such as ATP. In agreement with this, high serum phosphate has recently been associated with lung and skin tumorigenesis in mouse models [1]. Although this example seems striking, the influence of phosphate on cancer cell proliferation has not been characterized to date. Elucidating the role of phosphate in cancer metabolism will be fundamental in understanding the needs of tumor cells. This is particularly relevant in breast cancer and bone metastasis, as bone is the single largest source of stored phosphorus. Additionally, the high local phosphate concentration during osteolysis represents a potential factor contributing to the cell’s prolific microenvironment. Taking an interdisciplinary approach combining cell biology and metabolomics will help us understand how the metabolic network can be rewired in response to changes in phosphate availability.

## Materials and methods

Global metabolome analysis was conducted on a panel of breast cancer cell lines including MDA-MB-231 and MDA-MB-468 cultured with increasing phosphate levels, ranging from 1mM to 20mM. Metabolite extraction was performed using cold methanol and samples were analyzed on an orbitrap based LC-MS. Metabolite levels were characterized using an established database.

## Results

The relative abundance of numerous metabolites changed significantly. These include NADPH, which decreased by approximately 80-fold and FMN, which increased approximately 15-fold as phosphate was increased. Additionally, several glycolytic intermediates were observed to increase reproducibly in the presence of high phosphate. Currently, NADPH turnover is being further analyzed using stable isotope tracers. Furthermore, global metabolomics experiments under phosphate starvation conditions are being performed to identify additional metabolic changes and to corroborate if effects observed in a high phosphate context present a reciprocal effect in phosphate starvation. Taken together, changes observed in metabolism and cell behavior support the rationale that phosphate could indeed modulate the activity of cancer cells.
